# Evaluating breast cancer websites targeting Arabic speakers: empirical investigation of popularity, availability, accessibility, readability, and quality

**DOI:** 10.1186/s12911-022-01868-9

**Published:** 2022-05-09

**Authors:** Zahraa Jasem, Zainab AlMeraj, Dari Alhuwail

**Affiliations:** 1 Information Science Department, College of Life Sciences, Kuwait University, Kuwait City, Kuwait; 2 Health Informatics Unit, Dasman Diabetes Institute, Kuwait City, Kuwait

**Keywords:** Arabic, Availability, Breast cancer, Google trends, Middle East and North Africa, Popularity quality, Readability, WCAG, Website evaluation

## Abstract

**Background:**

Nowadays, patients have access to all types of health information on the internet, influencing their decision-making process. The Middle East and North Africa (MENA) region consists of 22 countries with an estimated population of around 600 million. Breast cancer is the highest diagnosed cancer in this region. Websites are commonly the go-to cancer information sources. A large population of the MENA region is only fluent in the Arabic language, thus access to Arabic websites is in more demand. However, little is known about breast cancer websites that cater to an Arabic-speaking audience. This study aims at evaluating Arabic breast cancer websites and offering recommendations to improve engagement and access to health information.

**Methods:**

This study employed a cross-sectional analysis approach. Google trends was used to reveal the top searched topics across the MENA region, which in turn were used as search terms to identify the websites. To be included, a website had to be active, available in Arabic, and contain breast cancer information. The evaluation was based on a combination of automated and expert-based evaluation methods through five dimensions: Availability, Accessibility, Readability, Quality, and Popularity.

**Results:**

Overall most of the websites performed poorly in the five dimensions and require careful reassessment concerning design, content, and readability levels; Only one website performed well in all dimensions, except for readability. Generally, the readability scores indicated that the websites were above the recommended level of reading. None of the websites passed the automated accessibility tests. The expert evaluation using the “Health on the Net” checklist showed good results for most websites.

**Conclusions:**

Breast cancer rates are rising in the MENA region, therefore having comprehensive, accurate, trustworthy, and easy-to-understand health information in their native language is a must. The results from this study show a need for improving the accessibility to breast cancer information websites available to Arabic speakers. The search was limited to three search engines yielding 10 websites and only one tool was used per dimension. Future work is needed to overcome these limitations. Collaboration between multiple stakeholders is necessary to develop websites that contain easy-to-read and understand high-quality information.

## Background

The World Wide Web has changed the way consumers seek health information challenging the concept of healthcare providers being the only reliable source of medical information [1]. Nowadays, patients have access to all types of health information through the internet [2]; for many, it is considered the first source of health information [3]. The percentage of people using the internet to obtain health-related information has increased in the past years, affecting the decision-making process for many patients and consumers [4]. In the United States alone, 72% of internet users seek health information about specific conditions, while in Europe it reached 71% [5].

Breast cancer is the most common cancer among women [6] and is the second leading cause of death among women in the United States [7] and is ranked number one globally [8]. The percentage of breast cancer patients seeking online information has reached 73% [9]. These patients use the internet to verify what information they are given, search for alternative treatments, and seek information related to cancer symptoms and treatment side effects [10, 11]. Patients use the internet as a source of information for many reasons, such as the sensitivity of the situation, mistrust of the healthcare system, and not having enough time with their healthcare providers [12].

For the past 10 years, cancer has been a rising problem in the Middle East and North Africa (MENA) region [13], which consists of 22 countries from Asia and Africa with an estimated population of around 600 million [14] or around 5.5% of the world’s population [15]. Breast cancer is the highest diagnosed cancer (17.7–19% of all types of cancer) [16] and accounts for 30% of female cancer [17]. Concerns have been raised due to the big increase in the percentage of breast cancer in the MENA region [18]. The lack of cancer education and barriers to cancer screening is seen as one of the major problems [19]. Education about this disease is important and will have positive effects on women’s practices, attitudes, and knowledge [20].

Medical and health websites can provide valuable sources for breast cancer information [4]. However, the unfiltered nature of the internet and the information it provides may disseminate misinformation and cause anxiety for patients [21]. Additionally, the quality of online information varies; missing or incorrect information may restrict patients from seeking appropriate care [22]. Moreover, the readability of medical terminologies and patient education materials has been reported difficult to read [23]. The accessibility of many health websites for people with special needs is limited, despite the increasing number of people with disabilities using assistive technologies [24].

The Arabic language, one of the official languages recognized by the United Nations [25], is spoken by a considerable percentage of the population in the MENA region; many are only fluent in the Arabic language, thus requiring access to Arabic content [12]. For online health information to be beneficial, the informational resources need to be organized, accessible, easily comprehended, and address patients’ specific needs [26]. Since little is known about cancer websites that cater to Arabic speakers, evaluating their availability, accessibility, readability, quality, and popularity becomes necessary to improve consumer engagement and access to health information for this population.

## Methods

This study employed a cross-sectional analysis approach [27, 28] to evaluate publicly- available Arabic breast cancer websites. The search for the websites was conducted within three weeks (15 Nov–8 Dec 2020) using the Google trends tool [29] and selected search engines for the 22 MENA countries. This was done to ensure that the included websites, including their pages, were not affected by their availability or major modifications.

### Google trends assessment

The Google Trends tool and selected search engines were used to retrieve information on breast cancer using related Arabic terms [30]. Google trends tool analyzes data from the Google search engine. It tracks keyword search queries users input to determine a search volume performed in a geographical region over a time range [31]. Relative search volume (RSV) is the representation of the data, i.e., the ratio between the total amount of Google queries and a specific topic. The values are displayed on a scale of 0–100, where 100 is the most popular, and 0 indicates not enough data for any one term. The higher the RSV the higher the term is searched [32, 33].

At the beginning of the search, the translated Arabic keyword of breast cancer “  ” was used as the search keyword for the period between Sept 1, 2018, and Nov 15, 2020. The selected period was intentional to ensure appropriate coverage of search terms over time and to reduce opportunities for any search biases due to particular events or trends. After that, the top popular topics revealed from the 22 MENA countries were counted and recorded. Finally, the top 10 topics were chosen as search terms and were used next in the search engines to identify the breast cancer websites.

### Selection of websites

1 were entered into the 3 most popular search engines “Google, Yahoo, and Bing” [24, 34, 35] on Dec 8, 2020, within 24 h to avoid any changes [36]. Only the three first search pages were evaluated in this study [24] as users don’t go beyond 3 pages when retrieving results while searching [34]. Inclusion and exclusion criteria were applied for the website’s selection [27, 37].

**Table 1:**
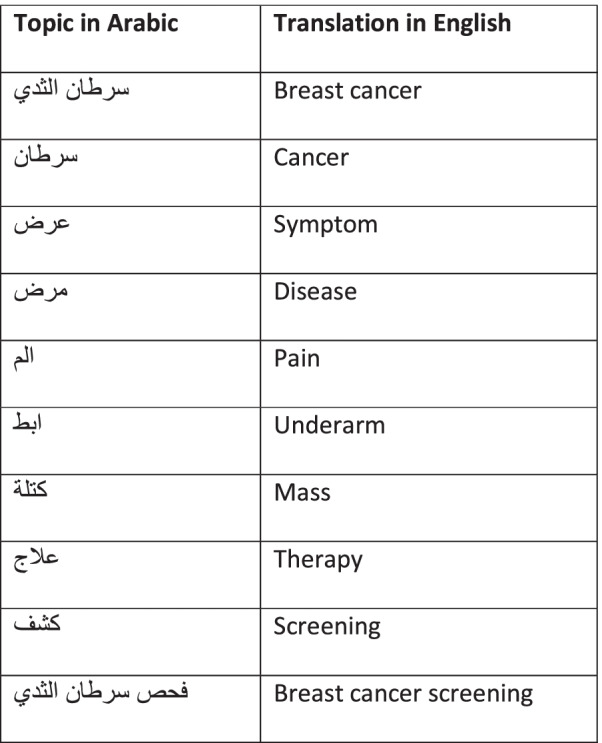
Topics in Arabic and their translation into English

### Inclusion/exclusion criteria

**Fig. 1:**
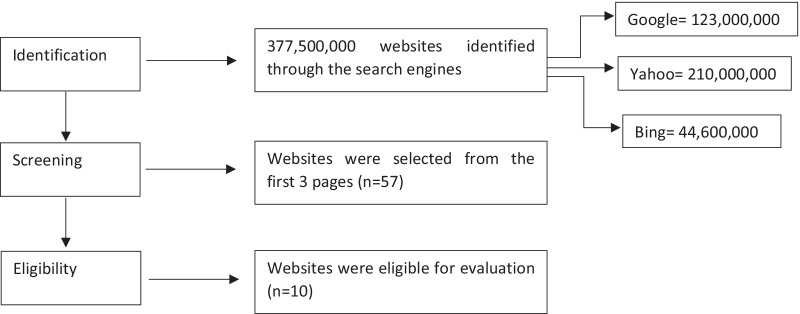
Flowchart depicting the website selection strategy

For websites to be included, they had to meet specific criteria: be active/reachable, available in the Arabic language, and contain breast cancer information. Websites were excluded if they were (1) duplicated; (2) In a language other than Arabic; (3) require ID and password for access; (4) mentioned breast cancer just by hints, audio, or visual-based; (5) Marked “Ad” in the search engine; and (6) were used purely for advertising or news. The search identified a total of 377,500,000 websites, and after applying the inclusion/exclusion criteria, 10 websites were eligible and assessed for Availability, Accessibility, Readability, and Quality. The different stages of the selection strategy are shown in Fig. 1.

### Evaluation

The following dimensions were evaluated in this study: Accessibility, Availability, Readability, Quality, and Popularity. Table 2 summarizes the tools and methods used to evaluate each dimension and whether it was checked automatically or by an expert [27]. These tools and methods were also used in similar studies, such as the accessibility evaluation of health websites [24, 27], the quality evaluation of other health websites [22], and the readability evaluation of online health information [38].

**Table 2:** Evaluation tools and methods used in this study to assess each dimension

Dimension	Tools and methods	Evaluation mode
Availability	Checklist of Google Trends analysis	Expert-based
Accessibility	Achecker [39] WAVE [40]	Automated Automated
Readability	“http://www.online-utility.org/english/readability_test_and_improve.jsp”	Automated
Quality	HON (Health on the Net) Checklist	Expert-based
Popularity	“https://www.prchecker.info/check_page_rank.php”	Automated

#### Availability

This dimension is an assessment of the website’s content availability. In the context of other studies [22], the evaluation involved assessing the availability of the top 10 topics according to Google Trends. Going through the websites and using the find search to indicate the existence of related information on the topics was used. All topics were searched in Arabic. “Breast cancer”, “cancer”, and “Disease” were combined as one, as well as “Screening” and “Detection”. This combination made the evaluation out of 7 topics instead of 10. The topics “Breast cancer, cancer, and disease” were considered available if a definition of breast cancer was mentioned. Then mentions of “Symptoms and therapy” were sought. Finally, the remaining topics as shown in Table 3 had to be mentioned at least once to be tallied.

**Table 3:**
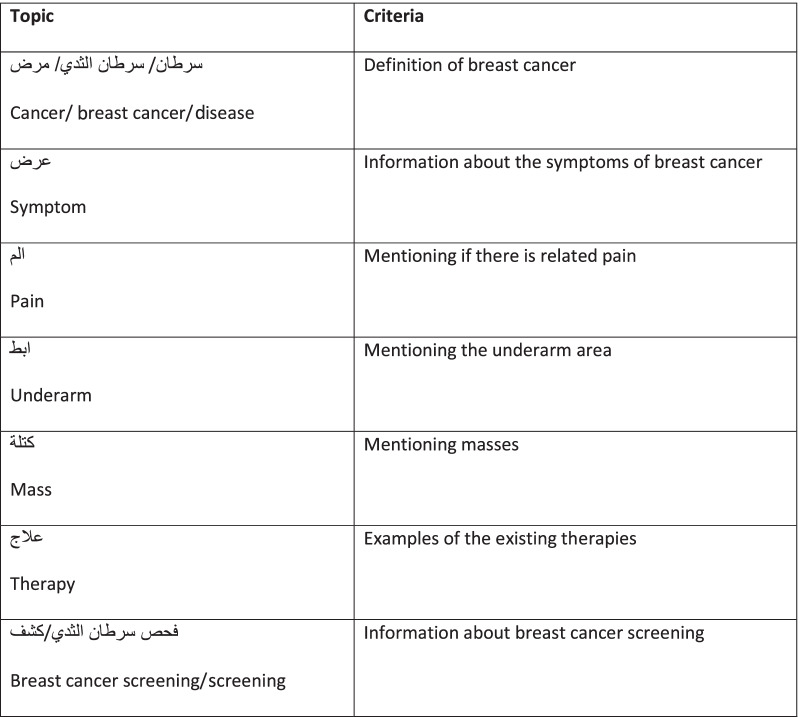
Availability evaluation criteria

#### Accessibility

To ensure that websites are correctly designed and developed to provide all users with equal access to information, an assessment of the website’s digital accessibility according to the World Wide Web Consortium (W3C) was conducted [41]. This is important because accessibility targets obstacles that prevent people with disabilities to interact with or access websites. The Web Content Accessibility Guidelines (WCAG) [42] content WCAG 2.0 at level AA was chosen as assessment criteria for this study, as it is the accepted level of performance worldwide [43]. The pages found on the search engines were assessed for this dimension.

The websites were evaluated using two automated tools Achecker and WAVE [41] to assess the following principles: Perceivable, Operable, Understandable, and Robust. Achecker [39] is a reliable tool that has been introduced in the W3C portal “*Web Accessibility Evaluation Tools List*” [24], it is free and has been used in many studies [27, 44, 45]. The tool generates reports about accessibility issues and divides them based on three categories:

(K) Known problems: These obstacles prevent accessibility
(L) Likely problems: These are probable obstacles requiring a human being to decide.
(P) Potential problems: These are problems that cannot be identified by the tool and require human judgment.

The second tool, WAVE [40], is another automatic tool developed by WebAIM that identifies ways to make a webpage more accessible to people with disabilities and has been used in many studies [43, 46, 47]. The tool checks for accessibility problems and divides them into six categories: errors, alerts, features, structural elements, contrast errors, and HTML5 and accessible rich internet application (ARIA).

#### Readability

This dimension is an assessment of the readability level of each website. Readability is “the ease with which written materials are read” [48] and is an influential factor in assessing the patients’ understanding of the written materials [49]. The readability level was assessed using an online tool, “*Readability Calculator*”^1^ which was used by similar other studies that analyzed Arabic websites [30, 50, 51]. The tool analyzes English text, as well as other languages as stated on the website, and is validated for Arabic text.

The Gunning Fog Index (GFI), the Coleman Liau Index (CLI), the Flesch Kincaid grade level (FKGL), the automated readability index (ARI), the simple measure of gobbledygook (SMOG), and the Flesch reading ease (FRE) were analyzed by the tool. However, only the FKGL, SMOG, and FRE were adopted for this study. The other indices are not suitable for the Arabic language since they count the number of letters, and the Arabic written language is comprised of words made up of letters that are linked to each other, not like in English.

The FRE is a 100-point scale, the higher the scores the more easily understood text, the scoring is shown in Table 4. While the FKGL represents the US education grade level. For example, a score of 7 indicates that the text is understood by those with 7 years of education. The recommended level for health materials is to be written at the 6th-grade level [21]. The SMOG represents the number of multi-syllabic words, the higher the words the higher the score [52]. FKGL and SMOG are contrary to FRE, the higher they are the more difficult the text is to understand. For the readability level to be satisfied the FRE was set to be ≥ 80.0, and < 7 for the FKGL and SMOG [21, 30, 49, 50].

**Table 4:** FRE scoring meaning

Score	Meaning
90–100	Very easy
80–89	Quite easy
70–79	Easy
60–69	Standard
50–59	Quite difficult
30–49	Difficult
0–29	Very complicated

#### Quality

This dimension is an assessment of a website’s quality using the Health On the Net (HON’s) websites evaluation checklist [27, 53]. HON is a non-profit institution that aims to assess the quality and transparency of data through eight principles: authority, complementarity, confidentiality, justifiability, attribution, financial disclosure, transparency, and advertising policy [27, 36, 54]. This study evaluated these principles by an expert walkthrough method. The pages found on the search engines, the home page, contact page, and the about us page were assessed for this dimension. Two experts evaluated the websites, and any disagreements were solved through discussions. The Financial principle was excluded from the assessment due to the difficulty of locating such data on the evaluated websites.

#### Popularity

This dimension assesses the ranking of the websites using an online tool called PR Checker^2^ similar to other studies [50, 55–57]. The tool was used to analyze Google’s PageRank, which calculates the amount and quality of links to a page to determine how important the website is. The scoring ranges from 0 to 10, where 10 is the better extreme. The most-visited websites have a PR of 10, while the least-visited websites have a PR of zero. A good page rank ranges from 5 and above [57].

## Results

### Availability of topics

**Fig. 2:**
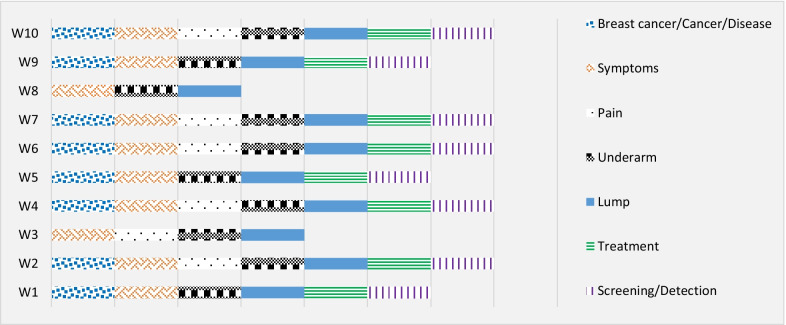
The assessed topics mentioned across all 10 websites

The results of this evaluation revealed that 50% of the websites had fulfilled the mentioning of all the target topics. As mentioned in the methods section, the assessment is out of 7 topics combining topics breast cancer, cancer, and disease as well as screening with detection. Websites (W1, W5, W9) were missing the mention of only one topic, while website (W3) was missing two, and finally, website (W8) had the worst result with mentioning only three of the topic criteria. Fig. 2 illustrates each website and the criteria of the topics.

### Accessibility

This evaluation was examined through two tools: Achecker and WAVE, according to WCAG 2.0 standards Level AA accessibility guidelines. The results of each evaluation are described below:

#### Achecker

The results of this tool point out that there was only one website (W8) that passed the evaluation. Website (W4) had good performance with only 2 known problems. Five websites (W1, W2, W3, W4, W7, W9) had known problems (39, 19, 89, 2, 40, 47) below average (the average = 121) while (W5, W6, W10) websites were with scores (130, 703, 138) above average. Table 5 shows the number of identified problems Known, Likely, and Potential across all 10 web pages found in the search engines. The tool identified 1207 known problems, 43 likely problems, and 9270 potential problems for all the websites. Noteworthy is the impact of “known problems” and how easily they can be solved. A description of these problems is summarized in *P = *Pass, F* = *Failed.*

**Table 5:** Achecker webpage evaluation results for level AA

Website ID	Result*	Known problems	Likely problems	Potential problems
W1	F	39	14	918
W2	F	19	13	1168
W3	F	89	0	649
W4	F	2	1	536
W5	F	130	0	699
W6	F	703	0	2602
W7	F	40	0	818
W8	P	0	0	0
W9	F	47	2	464
W10	F	138	13	1416

*P = Pass, *F* = Failed

Tables 6 and 7 summarizes the known problems according to the POUR principles, which show how high the number of perceivable errors is compared to the others. These 1110 perceivable errors generate obstacles for people using assistive technologies. In second place comes the operable errors with a total of 60, which mainly impact keyboard users. In third place comes the understandable errors with a total of 36 errors causing various obstacles for all users ranging from language to functionalities. Finally comes the robust errors with a positive performance of only 4 errors, indicating that the webpages work well across different platforms, technologies, and devices.

**Table 6:** A list of the most commonly known accessibility problems for Achecker analysis

List of commonly known problems (Level AA)	Count	Category
Element “B” or (bold) used	494	Perceivable
Element “i” or italic used	467	Perceivable
Image used as anchor is missing valid “alt” text	70	Perceivable
Anchor contains no text	53	Operable
Label text is empty	31	Understandable
Element “img” missing “alt” attribute	15	Perceivable
Input element type of “text” has no/missing associated label	15	Perceivable
Input element type of “text” has no text in label	12	Perceivable
Insufficient contrast between text color and its background	7	Perceivable
Input element, type of “checkbox”, has no text in label	6	Perceivable
Header nesting error	5	Operable
Text area element missing an associated label	5	Perceivable
Data table with more than one row/column of headers does not use id and headers attributes to identify cells	5	Perceivable
Element selected missing an associated label	4	Perceivable
Id attribute is not unique	4	Robust
Input element, type of “checkbox”, missing an associated label	4	Perceivable
On-mouseover event handler missing on-focus event handler	1	Operable
Script not keyboard accessible—on-mouse-out missing on-blur	1	Operable
Document language not identified	1	Understandable
Document has invalid language code	1	Understandable
Input element has alt attribute	1	Perceivable
Right to left reading order not marked or marked incorrectly	1	Understandable
Input element has more than one associated label	1	Perceivable

**Table 7:** The known problems according to POUR principles for Achecker analysis

ID	Perceivable	Operable	Understandable	Robust
W1	36	2	1	0
W2	8	4	6	1
W3	75	7	7	0
W4	1	1	0	0
W5	122	0	7	1
W6	666	34	3	1
W7	38	0	2	0
W8	0	0	0	0
W9	39	0	8	0
W10	125	12	2	1

#### WAVE

The WAVE tool evaluates the accessibility conformance through six categories: errors, alerts, features, structural elements, HTML5 and ARIA, and contrast errors. Table 8 summarizes this evaluation, showing that none of the websites passed the accessibility test. Website (W8) had the best performance with the least number of errors. The website (W10) had an invalid URL and was excluded from the analysis. Three websites (W3, W6, W7) had errors [21, 27, 47] above the average (the average = 16), while the rest had errors that were below and within the average. Notable is the low number of errors of all web pages compared to the other categories and how easily they can be solved.

**Table 8:** WAVE webpage evaluation results for level AA

ID	Errors	Alerts	Structural elements	HTML5 and ARIA	Features	Contrast errors
W1	16	46	45	10	59	0
W2	14	40	158	35	87	64
W3	27	101	52	74	38	112
W4	8	4	68	83	11	0
W5	8	36	75	0	25	45
W6	47	405	225	16	103	6
W7	21	91	90	32	13	72
W8	5	90	98	32	32	97
W9	16	9	53	23	11	14
W10	Bad request—invalid URL*	Bad request—invalid URL*	Bad request—invalid URL*	Bad request—invalid URL*	Bad request—invalid URL*	Bad request—invalid URL*
Total	162	822	864	305	379	410

*Bad request—invalid URL: the website did not open due to a request that hasn’t been met successfully

Table 9 summarizes the type of errors detected by the WAVE tool. The highest number of errors detected were “Very low contrast” i.e., low contrast between text and background colors. This can be resolved by setting the contrast ratio for foreground text versus background to at least 4.5:1, and at 3:1 for larger text [43, 58]. In the following positions, “Linked image missing alternative text”, “Missing form label”, “Empty link”, and “Language missing or invalid” errors which cause accessibility problems to screen-readers. By fixing these types of errors, websites will be accessible to keyboard and screen-reader users.

**Table 9:** The WAVE tool error analysis

Error	What it means	Count
Very low contrast	Very low contrast between text and background colors	410
Linked image missing alternative text	An image without alternative text results in an empty link	67
Missing form label	A form control does not have a corresponding label	30
Empty link	A link contains no text	23
Language missing or invalid	The language of the document is not identified or a lang attribute value is invalid	13
Broken ARIA menu	An ARIA menu does not contain required menu items	10
Missing alternative text	Image alternative text is not present	7
Empty button	A button is empty or has no value text	6
Empty form label	A form label is present but does not contain any content	3
Empty heading	A heading contains no content	2
Spacer image missing alternative text	A layout spacer image (which should have null/empty alternative text) does not have an alt attribute	1
Multiple form labels	A form control has more than one label associated with it	1

Table 10 summarizes the errors according to the POUR principles, which shows how high the number of perceivable errors is compared to the others. As mentioned in the Achecker section, the 561 perceivable errors are the biggest obstacles for people using assistive technologies. This is followed by operable errors (143 errors), understandable errors (38 errors), and finally robust errors (10 errors). Web developers should pay attention to these errors which cause accessibility limitations to a large number of users especially people with disabilities.

**Table 10:** The errors according to POUR principles for WAVE analysis

ID	Perceivable	Operable	Understandable	Robust
W1	14	12	0	0
W2	68	14	2	1
W3	145	25	15	2
W4	2	10	0	6
W5	56	6	5	0
W6	42	35	13	0
W7	94	20	2	0
W8	101	5	0	1
W9	39	16	10	0
W10	Bad request—invalid URL*	Bad request—invalid URL*	Bad request—invalid URL*	Bad request—invalid URL*

*Bad request—invalid URL: the website did not open due to a request that hasn’t been met successfully

**Fig. 3:**
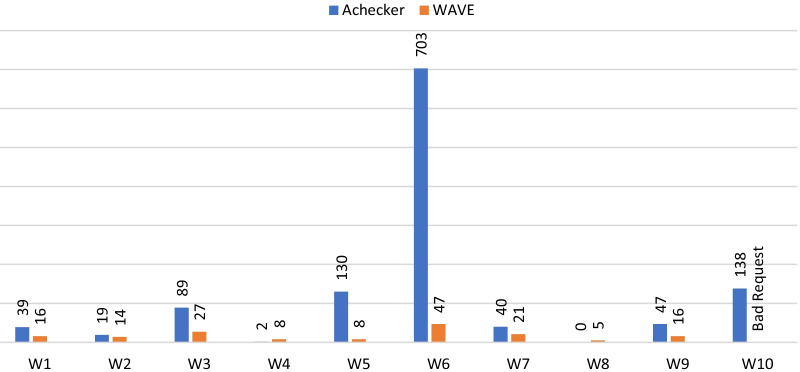
The number of known problems and errors of Achecker and WAVE for the websites

Fig. 3 illustrates the differences in results found between the Achecker and WAVE tools. The number of known problems and errors has a larger impact on the accessibility of websites. In comparison, it is shown that website (W8) had the best performance followed by website (W4). From both analyses websites (W1, W2, W4, W9) had problems below average in both tools, and websites (W3, W6) had problems above average in both tools. When combining the results of the two analyses none of the websites passed the accessibility test.

### Readability

The results of the readability analysis showed that according to the Flesch Kincaid grade level only 30% (W3, W5, W7) of the included websites had the recommended score level of a sixth grade and below, while 70% of the websites had a score above the seventh grade (≥ 7). None of the websites passed the Flesch reading ease score. The median grade level of the FRE was 58.05 and at the eighth-grade level according to the FKGL which indicates a challenging level of reading. More details are shown in Table 11. The most difficult website to read was (W1) with an FRE score of 12.33. Table 12 shows the detailed assessments of each website. According to the SMOG index score, all websites had a score above or equal to 7, which also indicates that it’s a difficult reading level.

**Table 11:** Readability analysis of the websites

	Flesch kincaid grade	SMOG	Flesch reading ease
Mean	8.48	9.38	52.55
SD	2.65	1.5	16.25
RSD	0.31	0.16	0.3
Median	8.14	9.36	58.05
Min	4.79	7.72	12.33
Max	13.70	11.71	71.89
25–75 IQ	6.4–10.55	7.98–10.33	45.78–59.93
< 7 score	30% (n = 3)	0% (n = 0)	NA*
≥ 7 score	70% (n = 7)	100% (n = 10)	NA*
≥ 80 score	NA*	NA*	0% (n = 0)
< 80 score	NA*	NA*	100% (n = 10)

*NA not applicable

**Table 12:** FRE score and FKGL level for each website

ID	Reading ease (FRE)*	Grade (FKGL)
W1	12.33	13
W2	57.81	10
W3	71.89	4
W4	43.54	10
W5	62.45	6
W6	45.78	9
W7	59.04	6
W8	54.43	7
W9	58.30	7
W10	59.93	8

*(100–90 very easy, 89–80 quite easy, 79–70 easy, 69–60 standard, 59–50 quite difficult, 49–30 difficult, 29–0 very complicated)

### Quality

The results of the HON expert analysis as indicated in Table 13, shows that only 2 websites (W2 and W10) passed the test with a score of 100% in all principles, websites (W7, W8, W9) had scores (14.28%, 42.84%, 14.28%) below average (the average = 65.6%) and the remaining 5 websites (W1, W3, W4, W5, W6) had high scores (71.4%, 71.4%, 85.68%, 71.4%, 85.68%) above average. While the results of the automated HON code seal^3^ were valid for only 2 websites (W4 and W10). The principles of Justifiability, Transparency, and Confidentiality were the highest fulfilled principles across all websites (n = 8), after that came Authority and Attribution principles (n = 7). While the least fulfilled principle was Complementarity (n = 3).

**Table 13:** Results of the HON expert analysis

ID	Results of HON code seal	Authority (14.285%)	Complementarity (14.285%)	Confidentiality (14.285%)	Attribution (14.285%)	Justifiability (14.285%)	Transparency (14.285%)	Financial disclosure (xclude)	Advertisement policy (14.285%)	Results of expert analyzing (100%)
W1	F	P	NP	P	P	NP	P	Excluded	Don’t apply	71.4%
W2	F	P	P	P	P	P	P	Excluded	Don’t apply	100%
W3	F	P	NP	P	P	P	P	Excluded	NP	71.4%
W4	P	P	NP	P	P	P	P	Excluded	P	85.68%
W5	F	P	P	P	NP	P	P	Excluded	NP	71.4%
W6	F	P	NP	P	P	P	P	Excluded	P	85.68%
W7	F	NP	NP	NP	NP	P	NP	Excluded	NP	14.28%
W8	F	NP	NP	P	P	NP	P	Excluded	NP	42.84%
W9	F	NP	NP	NP	NP	P	NP	Excluded	NP	14.28%
W10	P	P	P	P	P	P	P	Excluded	Don’t apply	100%

*Pass = P, Not Pass = NP

#### Editorial team

The majority of the websites (n = 7) had information about the site's team and only (n = 3) of the websites (W7, W8, W9) didn’t mention any authority information.

#### Complementarity

More than half of the websites (n = 7) didn’t mention a clear state that the information does not replace the relationship between the physician and patient.

#### Confidentiality of personal data

The privacy policy of data collection, storage, third parties, use of cookies and google analytics was mentioned in (n = 8) of the websites.

#### Attribution

A large number of the websites (n = 7) had listed the date of the last update of the medical information.

#### Justifiability

The health information on (n = 8) of the websites was provided in an objective, balanced and transparent manner. Sites with treatments had information concerning contraindications, adverse reactions, interactions, and precautions.

#### Transparency

Only (n = 2) of the websites did not have a contact e-mail address or contact form, the remaining (n = 8) were easy to use, their mission was clear, and they can be easily contacted.

#### Advertisement policy

Three of the websites did not include advertisements in their sites, while half of the websites didn’t clearly state the advertisements with the term “advertising”, remaining only (n = 2) of the websites fulfilling this principle as recommended by the HON Foundation.

#### Popularity

The results of Google page ranking shown in Table 14, showed that four websites (W1, W4, W6, W8) had scores ≥ 5, four websites (W2, W3, W5, W7) had scores below 5, and two websites (W9, W10) had no scoring due to being the least-visited websites on the search engine.

**Table 14:** Websites’ Google page ranking

Website ID	Rank /10
W1	7
W2	4
W3	4
W4	7
W5	3
W6	7
W7	4
W8	5
W9	0
W10	0

## Discussion

Consumers, patients, and caregivers are increasingly using the internet to seek breast cancer information as well as other health-related information [38]. Many studies have investigated the accessibility, availability, quality, and readability of online breast cancer information in various languages and none of their results met the recommended levels to ensure their effectiveness [38, 59–61]. Websites for breast cancer information with low quality, readability, and accessibility can lead to confusion, misconception, and limited access [62]. To the best of our knowledge, no prior study analyzed the popularity, accessibility, availability, quality, and readability of Arabic breast cancer information websites.

Overall, the evidence emerging from this study shows poor levels for most of the websites in the dimension discussed earlier. Only one website (W4) had good performance in all the evaluations except its readability test, which gave an FRE score < 80, and FKGL ≥ 7 indicating its reading level was hard for the average reader and above the recommended reading levels for health information. This website had high performance in accessibility and was associated with the HON code seal despite not passing the expert evaluation, it was also one of the highest scores by Google PageRank.

*Availability of content*—In general, half of the websites (50%) fulfilled the availability of content analysis, and this could differ from one study to another according to the selection of trending topics based on the selected area in a specific period.

*Accessibility—*Lately, website accessibility has received more attention than in the past. Adherence to guidelines for web accessibility ensures that people with disabilities and those abled people have access to the same information. Making websites accessible allows more people to use the internet for seeking health information, regardless of their disability. Online health information should be disseminated and accessed by a wide group of audiences [24, 46]. The present study shows that none of the websites passed the accessibility test (see Fig. 3). There were many violations of the POUR principles of WCAG 2.0, the highest was under the Perceivable principle, followed by operable, understandable, and robust respectively. This makes websites difficult to use by people with disabilities and is considered a form of discrimination by law and international conventions [63, 64].

The results from this study demonstrate that the most recurring violations were under the perceivable principle, meaning that the information is not being presentable in ways that all people can see or read, especially those using assistive technologies. Violations under the operable principle cause navigation difficulty for users while understandable violations are due to missing labels and language-related errors. Finally, robustness violations make it difficult to adapt to different user applications.

The findings from this study were in agreement with other studies, demonstrating that there are considerable barriers to receiving information on many public health websites throughout the world, which would mean that people with disabilities have inequitable access to health information online. [24, 65, 66]. Overall, this analysis highlights that accessibility standards are often overlooked across many websites, including breast cancer websites [24, 27, 65]. Web developers should be trained in accessibility and how to apply related standards and be aware of errors, especially those that can be fixed easily.

*Readability—*In terms of readability, more than half of the websites were found to be difficult to read, with scores above the recommended level of 7th grade. This means that the health information on the websites is difficult to understand and read by the general public [21, 67]. The National Institutes of Health recommends writing health-related materials in the sixth to seventh-grade level [68]. Unsurprisingly, the findings reported in this study are consistent with those of prior studies [21, 60–62, 67]. Websites have transitioned from static sources of information to dynamic applications that present a wide range of information [67].

The inability to read and understand the resources available can have a negative impact on the mental and physical health of cancer patients [51]. Websites should use easy-to-read language and avoid unnecessary difficult terminologies, especially when providing online health information.

*Quality—*In addition to readability, the quality of health websites is important, as it may affect the patients’ decision-making. The results of the expert assessment showed that 70% of the websites had scores above the average (average = 65.6%) including two websites scoring 100%. Since HONcode aims at promoting reliable, good-quality, transparent, and objective online health information [69], these findings indicate that the majority of websites were generally of good quality. Our results conflicted with other studies that focused on other health-related topics [30, 50, 54, 69]. This could be due to other studies relying on the automated HON code seal only. When comparing their results, our results will line up in terms of the HON code seal as only two of the websites had the seal.

*Popularity—*According to the Google ranking, only (40%) of the websites had good scoring. Although HON certification is associated with a higher ranking for the website [38], findings show no direct relation between the position in the Google ranking and a higher HON quality score. These findings could be due to the association between the automated HON certificate and the Google ranking [50], not the expert evaluator in this study. The lower ranking of the pages in the search engines reduces the probability of accessing high-quality medical information [70]. There could be opportunities for search engines to invest and use algorithms that promote websites with higher quality information.

### Study strengths and limitations

It is worth noting that there were some limitations in our study. The search was limited to three search engines and included the first three search pages as users usually don’t go beyond them [34]. While the internet has a vast amount of breast cancer content, only 10 websites that met the inclusion criteria and were on the top 3 pages of search results were considered for assessment. This might not necessarily reflect the status-quo of all online Arabic breast cancer websites, however, these websites were on the top 3 pages of search results, indicating they were very likely to be viewed by the public.

Furthermore, this study used free open-source automatic tools to evaluate the accessibility of the websites, however, using these tools was to achieve more reliable results [71]. Only the pages that were found during the search via search engines were the ones assessed, therefore it cannot be claimed that a website does not comply with accessibility standards by evaluating a single page. Adding human experts, including people with disabilities, in the evaluation and assessment of the accessibility of the websites would help achieve more reliable results. This study took a cross-sectional approach and evaluated the websites during a specific period, therefore the results may change as websites often get updated, changed, or retired. Future studies can build automated surveillance tools to assess accessibility. Finally, to test the quality, readability, and popularity dimensions, only one tool was used per dimension, which is consistent with similar earlier studies. However, it is imperative to note that no one tool is perfect. Future studies are needed to overcome these limitations.

## Conclusions

Nowadays, the Internet is a helpful tool for obtaining information about diseases, their prevention, and treatment approaches. This information should be taken from trustworthy, easy to access, and reliable resources. To our knowledge, this was the first study to evaluate the availability, accessibility, quality, readability, and popularity of online breast cancer websites in the Arabic language. The overall findings show that these websites had poor accessibility, low ranking on the internet, and are difficult to read by the general audience. Considering the growing number of breast cancer patients who use the internet to obtain medical information, having comprehensive, accurate, trustworthy, and easy-to-understand health information in their native language is a must. Urgent action must be taken to manage the websites that provide Arabic health information on breast cancer disease. Further work needs to be done to improve the quality and readability of online information for patients and to ensure that this information is also accessible. These websites can use the recommendations resulting from this study to improve their websites. Health professionals need to give recommendations and support the development of websites that are easy to read and contain high-quality information.

## Data Availability

All data generated or analyzed during this study are included in this published article [and its additional files].

## Abbreviations

MENA: The Middle East and North Africa
RSV: Relative search volume
FKGL: Flesch Kincaid grade level
SMOG: Simple measure of gobbledygook
FRE: Flesch reading ease
HON: Health On the Net

## Footnotes

1 https://www.online-utility.org/english/readability_test_and_improve.jsp.

2 https://www.prchecker.info/check_page_rank.php.

3 A stamp seal that is displayed on the website and is valid for one year to assure the fulfillment of the principles.
